# A Heptamethine Cyanine-Based Near-Infrared Optical Sensor for Copper(II) Detection in Aqueous Solutions and Living Cells

**DOI:** 10.3390/s26010130

**Published:** 2025-12-24

**Authors:** Ziya Aydin, Bing Yan, Maolin Guo

**Affiliations:** 1 Department of Chemistry and Biochemistry and UMass Cranberry Health Research Center, University of Massachusetts Dartmouth, 285 Old Westport Road, Dartmouth, MA 02747, USA; 2 Department of Chemistry, University of Massachusetts Amherst, 710 North Pleasant Street, Amherst, MA 01003, USA; 3 Vocational School of Technical Sciences, Karamanoğlu Mehmetbey University, 70200 Karaman, Türkiye; 4 Biomedical Engineering and Biotechnology Program, University of Massachusetts Dartmouth, 285 Old Westport Road, Dartmouth, MA 02747, USA

**Keywords:** optical sensor, near-infrared (NIR), fluorescent, copper detection, heptamethine cyanine, 1,10-phenanthroline, bio-imaging

## Abstract

Copper ions are essential trace elements that play critical roles in redox reactions, signal transduction, energy metabolism, and regulation of the central nervous system. However, excess copper can induce cytotoxicity and contribute to various pathological conditions, highlighting the need for sensitive and selective detection methods. We report a novel near-infrared (NIR) optical sensor, IRPhen, based on a heptamethine cyanine scaffold conjugated with a 1,10-phenanthroline Cu^2+^-binding receptor. IRPhen exhibits strong NIR absorption and emission (E_x_: 750 nm, E_m_: 808 nm), high sensitivity, and good selectivity toward Cu^2+^ over competing metal ions. Spectroscopic studies revealed a rapid, reversible 1:1 binding interaction with a binding constant of 1.3 × 10^6^ M^−1^ and a detection limit of 0.286 µM. The probe demonstrated excellent stability across physiological pH ranges and maintained its performance under competitive conditions. Importantly, IRPhen is cell-permeable and capable of detecting dynamic Cu^2+^ changes in living fibroblast (WS1) cells using confocal microscopy. This sensor design offers a versatile platform for developing NIR optical sensors to study copper homeostasis, elucidating copper-related biological mechanisms, and potentially developing similar NIR probes for other biologically relevant metal ions.

## 1. Introduction

As an essential element for life, copper plays a key role in numerous physiological and biochemical processes, including signal transduction, redox regulation, energy metabolism, and maintenance of the central nervous system [1,2,3]. Its ability to reversibly cycle between oxidized (Cu^2+^) and reduced (Cu^+^) states underlies its vital biological roles, particularly in enzymatic reactions involved in oxidative metabolism and antioxidative defense [3]. However, this same redox-active property also renders copper potentially toxic, as it can catalyze Fenton-like reactions that generate reactive oxygen species (ROS), leading to oxidative damage of nucleic acids, lipids, and proteins [3]. Maintaining copper homeostasis is therefore crucial for all living organisms. In humans, copper is the third most abundant essential trace metal after iron and zinc [2], with normal serum Cu^2+^ concentrations ranging from 15.7 to 23.6 µM. The human brain is particularly enriched in copper, containing approximately 9% of the body’s total copper content [4]. Dysregulation of copper metabolism is closely associated with a range of neurological and systemic disorders. Classical genetic diseases such as Menkes and Wilson’s diseases arise from impaired copper transport and storage [5], while copper dyshomeostasis is also implicated in common neurodegenerative conditions, including Alzheimer’s disease, Parkinson’s disease, Progressive Supranuclear Palsy, amyotrophic lateral sclerosis, and neuroblastoma [6,7].

Beyond its biological importance, copper is also widely used in various industrial sectors, including electrical wiring, machinery manufacturing, fertilizers, and batteries [4]. However, excessive copper exposure from industrial or environmental sources can cause environmental contamination, pollution and severe toxic effects in living organisms. Elevated Cu^2+^ levels have been linked to hepatic and renal injury, gastrointestinal dysfunction, hypoglycemia, and neonatal liver damage [5,6,7].Moreover, recent studies have identified a unique copper-dependent form of regulated cell death, termed cuproptosis, which results from the aggregation of lipoylated proteins and the loss of iron–sulfur cluster proteins [8]. These processes disrupt components of the tricarboxylic acid (TCA) cycle, causing proteotoxic stress and cell death [8].

Given copper’s dual nature as both an essential micronutrient and a potential cytotoxin, the development of sensitive and selective detection methods for Cu^2+^ is of critical importance. Such analytical tools are essential not only for monitoring copper pollution in environmental systems, but also for elucidating the molecular mechanisms by which copper homeostasis influences health and disease. Several analytical techniques have been developed to detect Cu^2+^ ions in environmental and biological samples, including atomic absorption spectroscopy (AAS), inductively coupled plasma mass spectrometry (ICP–MS), electrochemical analysis, and colorimetric or fluorescent assays [9,10,11,12,13,14,15]. While these methods offer high sensitivity and reliability, they often suffer from inherent limitations such as high instrumentation cost, complex sample pretreatment, and labor-intensive or sophisticated synthesis procedures. Consequently, there is growing interest in developing simple, cost-effective, and highly selective fluorescent sensors capable of real-time Cu^2+^ detection in complex biological and environmental systems.

Over the past few decades, fluorescent sensors have attracted increasing attention for the detection and visualization of metal ions in living systems [16,17,18,19]. However, most reported sensors exhibit absorption and emission wavelength in the UV-Vis region, which limits their suitability for in vivo imaging due to strong tissue autofluorescence, light scattering, and poor penetration depth. To overcome these drawbacks, near-infrared (NIR) fluorescent probes have emerged as a powerful alternative [20,21,22]. NIR fluorescence imaging, typically operating in the wavelength range of 650–900 nm (and extending up to 1350 nm in the so-called “second NIR window”), offers several significant advantages, including deeper tissue penetration, higher signal-to-noise ratios, and minimal background interference from biological components [23].

Despite these advantages, relatively few NIR-active Cu^2+^ fluorescent probes have been reported to date [24,25,26,27,28,29,30]. In most cases, the reported systems exhibit excitation wavelengths that remain in the UV or visible region, even though their fluorescence emissions extend into the short NIR range [31,32,33,34]. Only a small number of Cu^2+^ sensors feature both excitation and emission wavelengths that fall entirely within the true NIR window, which is essential for deep-tissue bioimaging and real-time monitoring of copper ions in living organisms [25,35].

Our group has recently developed a near-infrared fluorescent probe, CR-Ac (λ_ex_ = 650 nm, λ_em_ = 696 nm), for Cu^2+^ detection, based on a coumarin–benzopyrylium platform [36]. To further extend both excitation and emission into the longer NIR region, we sought to design new Cu^2+^ sensors using a heptamethine cyanine framework. Heptamethine cyanine dyes are well known for their strong absorption in the near-infrared region (650–900 nm), high molar extinction coefficients, low cytotoxicity, and tunable optical properties. Structural modifications in the polymethine chain or terminal heterocycles can significantly enhance their photostability, quantum yield, and chemical stability [37,38,39]. Notably, several derivatives have been approved by the U.S. Food and Drug Administration (FDA) as safe in vivo imaging agents, underscoring their biomedical potential. Heptamethine cyanine dyes have been approved by the FDA as in vivo imaging agents.

In this work, we report the design and synthesis of a novel NIR optical and fluorescent Cu^2+^ sensor, IRPhen, constructed by conjugating a Cu^2+^-binding unit, 1,10-phenanthroline, to a heptamethine cyanine scaffold. The resulting probe exhibits longer excitation and emission wavelengths, rapid fluorescence response, high sensitivity, and good selectivity toward Cu^2+^ ions. Furthermore, IRPhen demonstrates strong potential for Cu^2+^ detection in living cells, highlighting the potential of this designing strategy in developing novel NIR sensors for biological and environmental monitoring of metal ions.

## 2. Materials and Methods

### 2.1. Chemicals

5-Amino-1,10-phenanthroline, 4-hydroxybenzaldehyde, and IR-780 were purchased from Sigma-Aldrich (St. Louis, MO, USA)and used without further purification. All other reagents and solvents were of analytical grade, obtained commercially, and used as received. Unless otherwise specified, metal ion solutions were prepared from their respective nitrate or chloride salts in deionized water. A stock solution of IRPhen (500 µM) was prepared in acetonitrile (ACN) and diluted to 5 µM with an ACN/H_2_O mixture (*v*/*v* = 1:1) for spectroscopic measurements. Stock solutions of metal ions (10 mM) were prepared in deionized water using the chloride salts of Ni^2+^, Cu^+^, Cu^2+^, Zn^2+^, Fe^2+^, Fe^3+^, Cr^3+^, Hg^2+^, Mn^2+^, Ag^+^, Na^+^, and Ca^2+^, and the nitrate salts of Co^2+^, K^+^, Pb^2+^, and Mg^2+^. The Fe^2+^ and Fe^3+^ solutions were freshly prepared in 0.1 M HCl to minimize oxidation or hydrolysis, while the Cu^+^ solution was freshly prepared by dissolving tetrakis(acetonitrile)copper(I) hexafluorophosphate [Cu(CH_3_CN)_4_]PF_6_ (Sigma-Aldrich, St. Louis, MO, USA) in deionized water immediately prior to use.

### 2.2. Instrumentation and Spectroscopy

A Bruker (Billerica, MA, USA) DRX-300 NMR spectrometer was used to record ^1^H and ^13^C NMR spectra at 298 K. Chemical shifts are reported in δ (ppm) relative to tetramethylsilane (TMS) as an internal standard. Signal multiplicities are denoted as s (singlet), d (doublet), t (triplet), q (quartet), m (multiplet), and br (broad). Electrospray ionization mass spectrometry (ESI-MS) analyses were performed on a PerkinElmer (Waltham, MA, USA) API 150EX mass spectrometer. UV–Vis absorption spectra were recorded on a PerkinElmer Lambda 25 spectrophotometer at 298 K, and fluorescence measurements were conducted on a PerkinElmer LS55 luminescence spectrometer under the same temperature conditions. pH measurements were carried out using a Corning pH meter equipped with a Sigma-Aldrich micro-combination electrode, calibrated with standard buffer solutions. Cellular fluorescence imaging experiments were performed using a Zeiss LSM 710 laser scanning confocal microscope (Carl Zeiss, Oberkochen, Germany).

### 2.3. Synthesis and Characterization of IRPhen

The Cu^2+^-binding ligand, 4-(1,10-phenanthrolin-5-ylimino)methylphenol (Phen), was synthesized and characterized according to a previously reported procedure [40]. The Cu^2+^ sensor IRPhen was then prepared as follows. Under a nitrogen atmosphere, a slurry of NaH (60% in mineral oil, 15.2 mg 0.38 mmol) and DMF (1 mL) was added dropwise to a stirred solution of Phen (0.120 g, 0.4 mmol) in anhydrous DMF (2 mL) at 0 °C. After stirring for 30 min, the reaction mixture was allowed to warm to ambient temperature and subsequently added to a solution of IR-780 (200 mg, 0.30 mmol) in DMF (3 mL). The resulting mixture was stirred overnight, then quenched with water, and the solvent was removed under reduced pressure (below 40 °C) to afford a dark green solid crude product. The product was purified by silica gel column chromatography using (MeOH/DCM; 0/100 to 10/90, *v*/*v*) as the eluent to yield IRPhen as a dark green solid (40 mg, yield 15%).

^1^H NMR (300 MHz, CDCl_3_) δ (ppm): 9.15 (d, J = 4.0 Hz, 1H), 9.05 (d, J = 4.0 Hz, 1H), 8.84 (d, J = 6.5 Hz, 1H), 8.80 (s, 1H), 8.51(d, J = 6.5 Hz,1H), 8.22 (d, J = 8.0 Hz, 2H), 8.13 (m, 2H), 8.00 (d, J = 7.0 Hz, 2H), 7.79 (d, J = 11.3 Hz, 2H), 7.71 (d, J = 11 Hz, 1H), 7.54 (m, 2H), 7.51 (s, 1H), 7.48–7.10 (m, 7H, incl. solvent signals), 7.04 (d, J = 7.0 Hz, 2H), 6.96 (s, 1H), 6.11 (d, J=12 Hz, 2H), 4.18 (m, 4H), 3.62 (m, 4H), 3.17 (m, 4H), 2.06 (m, 2H), 1.96 (t, J = 6.9 Hz, 4H), 1.49 (s, 6H), 1.37 (m, 3H), 0.95 (s, 6H). ^13^C NMR (75 MHz, CDCl_3_) δ (ppm): 170.3, 167.84, 164.33, 154.54, 149.43, 147.0, 146.0, 141.6, 140.9, 139.0, 138.3, 137.0, 136.3, 135.0, 133.6, 132.0, 131.93, 130.4, 128.4, 125.3, 124.4, 123.3, 122.2, 121.8, 121.2, 120.6, 117.4, 114.5, 112.0, 111.0, 97.1, 52.4, 50.1, 49.2, 45.3, 28.1, 28.0, 26.9, 26.2, 23.2, 21.5, 20.4, 12.1, 11.4; ESI-MS (positive mode): *m/z* calc for C_55_H_56_ON_5_^+^, 802.4; found: 802.3.

### 2.4. Cell Culture and Confocal Imaging

Human skin primary fibroblast cells (WS1) purchased from American Type Culture Collection (ATCC, Manassas, VA, USA) were used in this study. The cells were cultured in Eagle’s Minimum Essential Medium supplemented with 10% fetal bovine serum (FBS) under standard conditions (37 °C, 5% CO_2_). Subculturing was performed using 0.25% trypsin-EDTA and was neutralized by adding complete growth medium. The cells were seeded into 25 cm^2^ flasks without centrifugation and the medium was replaced every two or three days until approximately 70% confluency was reached, after which the cells were transferred into Petri dishes for subsequent experiments.

A 10 mM stock solution of IRPhen was prepared in DMSO and diluted to 10 µM in culture medium without FBS. After replacing the culture medium with fresh serum-free medium, the cells were incubated with 10 µM of IRPhen. These cells were then subjected to fluorescence imaging using a Zeiss LSM710 confocal microscope. For Cu^2+^ co-incubation experiments, cells were first treated with 10 µM CuCl_2_ for 8 h, followed by incubation with 10 µM IRPhen for 30 min before confocal imaging.

## 3. Results and Discussion

### 3.1. Design and Synthesis of the Cu^2+^ Sensor IRPhen

A fluorescent sensor typically consists of two essential components: a recognition (binding) receptor and a fluorophore [41]. The 1,10-phenanthroline moiety, a well-established ligand with high affinity toward Cu^2+^, was selected as the recognition receptor. Owing to its intrinsic paramagnetic nature, Cu^2+^ efficiently quenches fluorescence, a property that has been widely utilized in the development of effective “turn-off” fluorescent sensors for Cu^2+^ detection [42,43]. Heptamethine cyanine dyes were chosen as the fluorophore platform due to their strong absorption in the near-infrared (NIR) region (650–900 nm), large molar extinction coefficients, low cytotoxicity, and tunable optical characteristics. Structural modification in heptamethine cyanine dyes allows for fine-tuning of their photophysical behavior, such as improving photostability and altering quantum yield, which are desirable features for bioimaging applications [37,38,39].

**Scheme 1:**
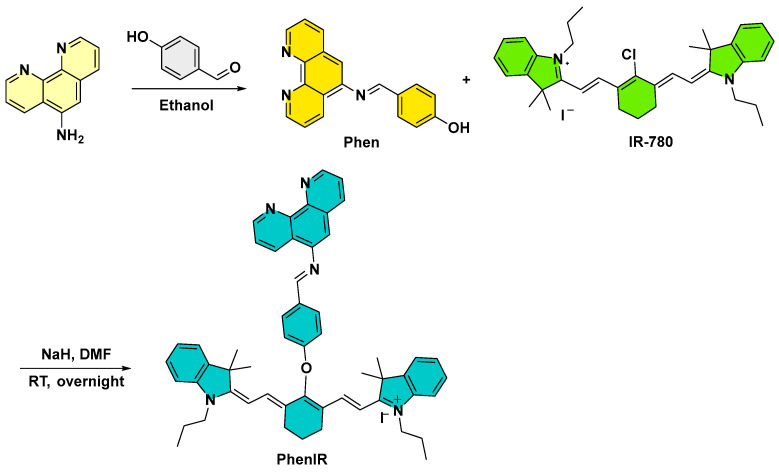
Synthetic scheme of IRPhen.

To construct the sensor, the 1,10-phenanthroline-based Cu^2+^-binding group was conjugated to the heptamethine cyanine (IR-780) dye through a methylphenol linker, yielding a novel near-infrared optical and turn-off fluorescent Cu^2+^ sensor, IRPhen. The sensor was synthesized using a straightforward 2-step procedure (Scheme 1) under mild conditions, affording an overall yield of 15%. The structure of the IRPhen sensor was fully characterized by NMR (^1^H NMR and ^13^C NMR) and ESI-MS mass spectrometry, confirming successful formation of the desired compound.

### 3.2. UV-Vis-NIR Absorption Spectra and Metal Ion Selectivity

The sensor has poor solubility in pure water-based solvents but is quite soluble in common polar organic solvents. To improve the solubility, a water/acetonitrile (ACN) mixture (1:1) was used as the solvent to perform the in vitro studies. Though the ACN/H_2_O mixture may not accurately reflect the natural states of copper ions and the sensor in a pure aqueous environment, the inside of a cell is not a dilute pure aqueous environment, but a crowded environment filled with high concentrations of macromolecules and many organic and inorganic small molecules. To better mimic this aspect, an ACN/H_2_O mixture (1:1) was chosen [36].

**Figure 1:**
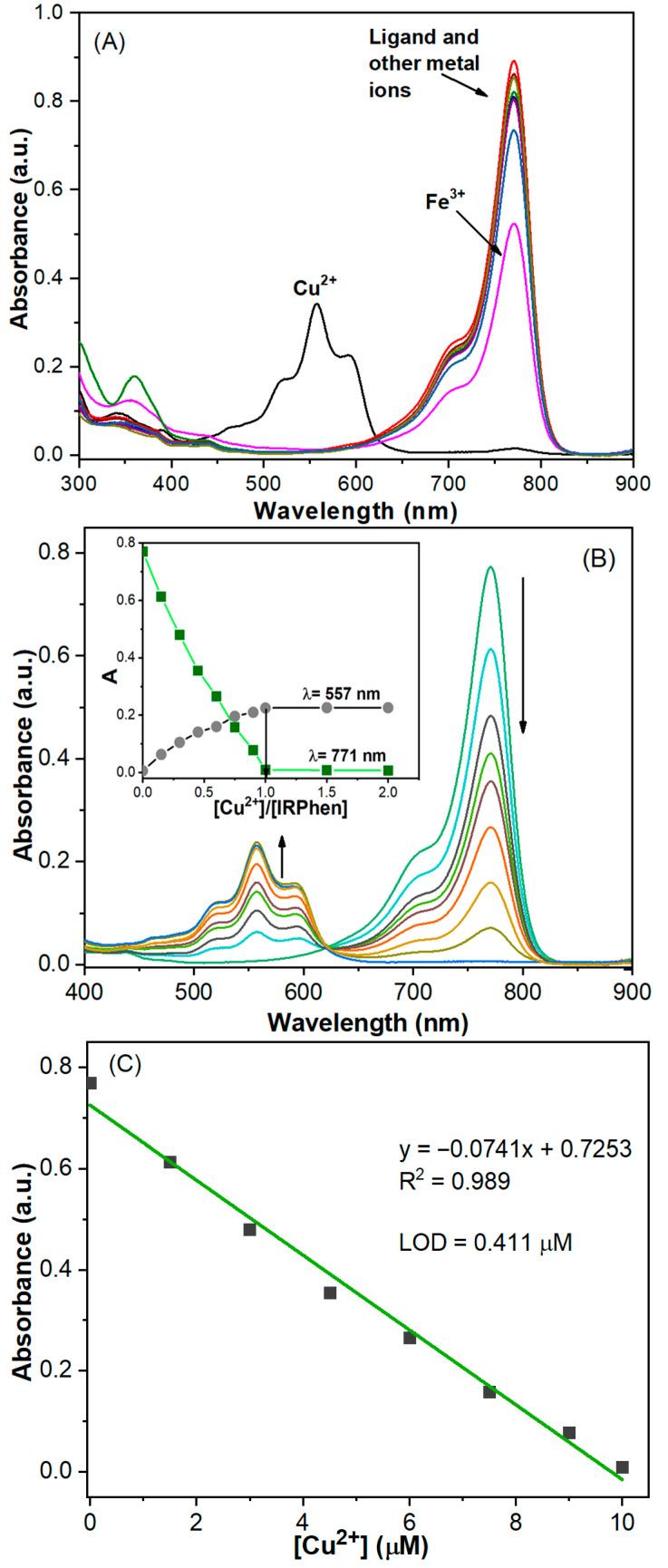
(**A**) UV-Vis-NIR absorption spectra of IRPhen (5 µM) in the presence of various metal ions (5 µM for Cu^2+^, Ni^2+^, Mn^2+^, Hg^2+^, Zn^2+^, Ag^+^, Pb^2+^, Co^2+^, Fe^3+^, Fe^2+^, Cu^+^ and Cr^3+^; 50 µM for Ca^2+^, Mg^2+^, K^+^ and Na^+^) in ACN/H_2_O (*v*/*v*. 1:1). (**B**) Absorption spectral changes in IRPhen (5 µM) upon gradual addition of CuCl_2_ (0, 0.25, 0.50, 1.0, 1.5, 2.0, 2.5, 3.0, 3.5, 4.0, 5.0, 7.5, and 10 µM, respectively) in ACN/H_2_O (*v*/*v*. 1:1). The letter “A” indicates absorbance and corresponds to the y-axis of the inside graph. (**C**) Linear relationship between absorbance intensity at 771 nm and Cu^2+^ concentration (0–10.0 µM) in ACN/H_2_O (*v*/*v*, 1:1) with [IRPhen] = 10.0 µM. The detection limit for Cu^2+^ was determined to be 0.411 µM by the absorbance method.

The UV-Vis-NIR absorption characteristics of IRPhen and its interactions with various metal ions were investigated in ACN/H_2_O (*v*/*v*, 1:1). As shown in Figure 1A, the solution of IRPhen alone (5.0 × 10^−6^ M) displayed a strong absorption peak in the NIR region with a maximum at 771 nm and a high molar coefficient (ε = 1.68 × 10^5^ M^−1^ cm^−1^), confirming its excellent NIR absorption properties.

The absorption spectral behavior of IRPhen upon interaction with various metal ions was investigated in ACN/H_2_O (*v*/*v*. 1:1) (Figure 1A). Upon the addition of Cu^2+^, the absorption band at 771 nm disappeared, while a new band centered at 557 nm with two shoulders, one at a shorter wavelength and one at a longer wavelength, simultaneously appeared. In contrast, other metal ions—including Cr^3+^, Cu^+^, Na^+^, Hg^2+^, Mg^2+^, Ca^2+^, Fe^3+^, Zn^2+^, Ag^+^, Pb^2+^, K^+^, Co^2+^, Fe^2+^, Mn^2+^, and Ni^2+^—produced negligible spectral changes under identical conditions. Only Fe^3+^ caused a slight decrease in the 771 nm band without generating a new absorption at 557 nm, indicating weak and nonspecific interaction. These results demonstrate that IRPhen exhibits excellent selectivity toward Cu^2+^ over a broad range of competing metal ions.

The spectral response of IRPhen to Cu^2+^ was further examined in detail (Figure 1B). Gradual addition of Cu^2+^ led to a complete decrease in the 771 nm band accompanied by a concurrent increase in the 557 nm band (ε = 4.16 × 10^4^ M^−1^ cm^−1^). This distinct hypsochromic (blue) shift is indicative of H-type aggregation of the cyanine dye, induced by Cu^2+^ coordination [44,45,46]. Moreover, the presence of a well-defined isosbestic point near 620 nm confirms a clean interconversion between the free and Cu^2+^-bound forms of IRPhen, consistent with a specific and stoichiometric binding process. Analysis of the linear relationship between absorbance intensity and Cu^2+^ concentration (0–10.0 µM) revealed a detection limit of 0.411 µM for Cu^2+^ in ACN/H_2_O (*v*/*v*, 1:1) (Figure 1C). Furthermore, the spectroscopic response to Cu^2+^ occurred almost instantaneously, indicating a rapid coordination event and fast sensing response.

### 3.3. Fluorescent Properties of IRPhen

**Figure 2:**
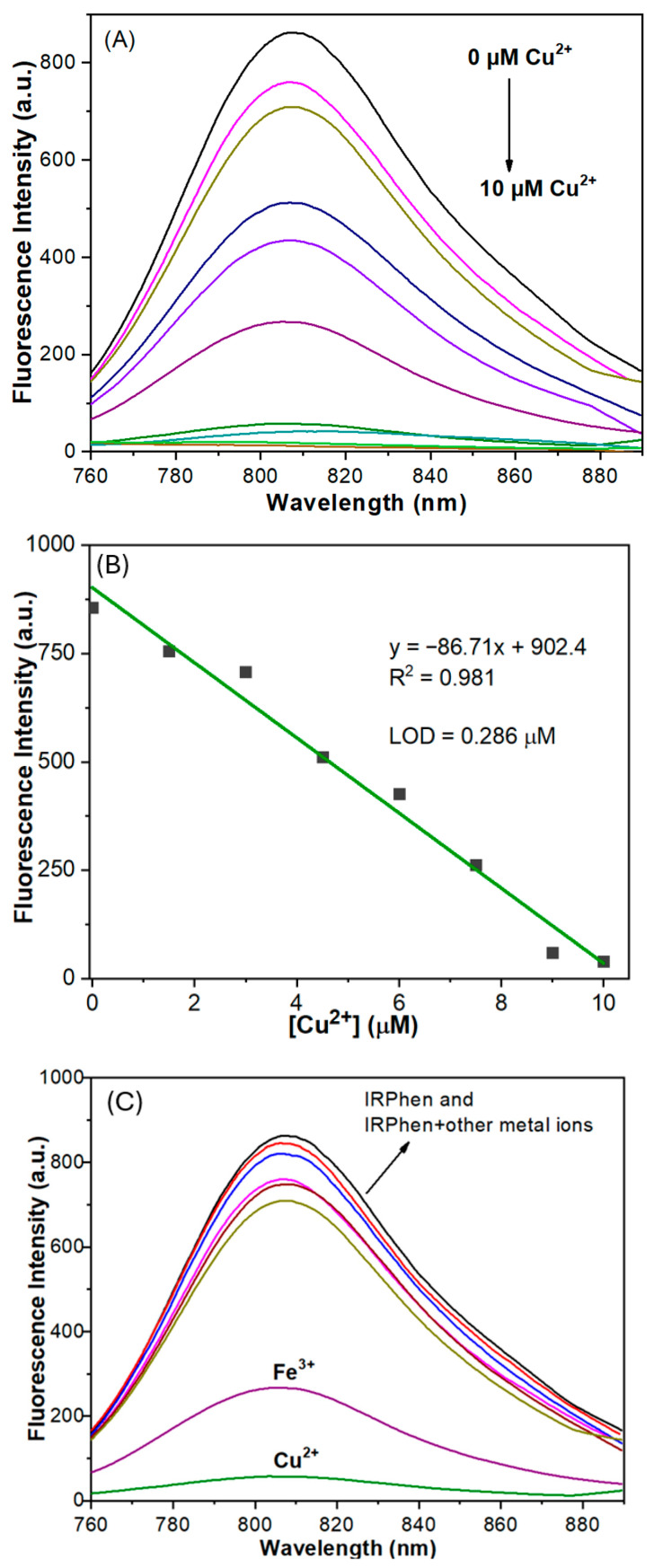
(**A**) Fluorescence spectra of IRPhen (5 µM) with gradual addition of CuCl_2_ (0, 0.25, 0.5, 1.0, 1.5, 2.0, 2.5, 3.0, 3.5, 4.0, 5.0, 7.5, and 10 µM) in ACN/H_2_O (*v*/*v*, 1:1). (**B**) Linear relationship between fluorescence intensity and Cu^2+^ concentration (0–10.0 µM) in ACN/H_2_O (*v*/*v*, 1:1) with [IRPhen] = 10.0 µM (Fluorescent intensities were recorded at an emission wavelength of 808 nm). The detection limit for Cu^2+^ was determined to be 0.286 µM by the fluorescent method. (**C**) Fluorescence spectra of IRPhen (5 µM) in the presence of various metal ions (5 µM for Cu^2+^, Hg^2+^, Mn^2+^, Ni^2+^, Zn^2+^, Ag^+^, Pb^2+^, Fe^3+^, Co^2+^, Fe^2+^, Cu^+^ and Cr^3+^; 50 µM for Ca^2+^, K^+^, Mg^2+^ and Na^+^) in ACN/H_2_O (*v*/*v*, 1:1).

When excited at 550 nm, IRPhen showed no emission in the 570–650 nm range, and only a very weak peak near 800 nm. In contrast, excitation at 750 nm produced a distinct emission peak at 808 nm (Figure 2A). To examine its fluorescence response to Cu^2+^, a solution of IRPhen in ACN/H_2_O mixture (*v*/*v*. 1:1) was titrated with increasing concentrations of Cu^2+^ and monitored by fluorometry at excitation wavelengths of 550 and 750 nm, respectively. The addition of Cu^2+^ to IRPhen had no effect on the fluorescence spectrum when excited at 550 nm; therefore, subsequent measurements were performed with excitation at 750 nm. Upon addition of Cu^2+^, the fluorescent intensity of 808 nm emission band decreased progressively, leading to complete quenching at 1 equiv. of copper ion (Figure 2A). This quenching behavior is attributed to the intrinsic paramagnetic nature of Cu^2+^ in the specific Cu^2+^-IRPhen complex. The coordination of paramagnetic Cu^2+^ ions (d^9^ electronic configuration with one unpaired electron) to 1,10-phenanthroline derivatives induces significant fluorescence quenching, a phenomenon reported in prior studies [47,48]. This fluorescence attenuation is attributed to contributions from both static (complex formation) and dynamic quenching (collisional deactivation) mechanisms within the resulting Cu^2+^-coordinated complexes [42,43,47,48]. The paramagnetic Cu^2+^ ions can drastically increase the rate of internal deactivation pathways (e.g., enhanced intersystem crossing, facilitation of non-radiative decay and photoinduced electron transfer), effectively “turning off” the fluorescence signal. Analysis of the linear relationship between fluorescence intensity and Cu^2+^ concentration (0–10.0 µM) yielded a detection limit of 0.286 µM for Cu^2+^ in ACN/H_2_O (*v*/*v*, 1:1) (Figure 2B). This result is attributed to the superior sensitivity of fluorescence spectroscopy, which provided a slightly better detection limit compared to the absorption measurement (0.411 µM) (Figure 1C).

In order to evaluate selectivity, the fluorescence response of IRPhen was examined in the presence of various metal ions, including Cr^3+^, Cu^+^, Na^+^, Hg^2+^, Mg^2+^, Ca^2+^, Fe^3+^, Zn^2+^, Ag^+^, Pb^2+^, K^+^, Co^2+^, Fe^2+^, Mn^2+^ and Ni^2+^. As shown in Figure 2C, only Cu^2+^ induced significant fluorescence quenching of IRPhen, while all other metal ions caused negligible spectral changes. A slight decrease in intensity was observed with Fe^3+^, likely due to its strong paramagnetic character and nonspecific interaction with the sensor.

**Figure 3:**
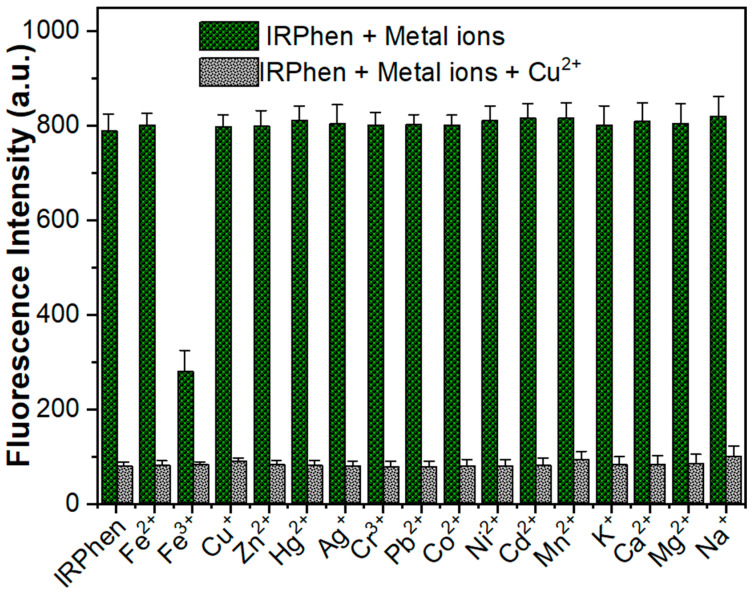
Fluorescence intensities of IRPhen (5 µM) with various metal ions (green bar) and after the subsequent addition of Cu^2+^ (gray bar) (Fluorescence intensities were recorded at an emission wavelength of 808 nm).

The detection of a target cation in the presence of other metal ions in a real sample is a critical assay for evaluating selectivity. To access the ability of IRPhen to resist interference from competing analytes during Cu^2+^ detection, a competition binding experiment was carried out. Various meal ions (Cr^3+^, Cu^+^, Na^+^, Hg^2+^, Mg^2+^, Ca^2+^, Fe^3+^, Zn^2+^, Ag^+^, Pb^2+^, K^+^, Co^2+^, Fe^2+^, Mn^2+^ and Ni^2+^) were first pre-incubated with IRPhen. As expected, no significant fluorescence change was observed (green bars in Figure 3) except for a slight decrease in intensity with Fe^3+^. However, upon subsequent addition of 1.0 equiv. of Cu^2+^ to each solution, pronounced fluorescence quenching occurred (gray bars in Figure 3). These results demonstrate that none of the tested metal ions interfere significantly with the Cu^2+^-specific sensing behavior of IRPhen, confirming its good selectivity toward Cu^2+^ even in competitive environments.

### 3.4. Binding Stoichiometry and Affinity Between IRPhen and Cu^2+^

**Figure 4:**
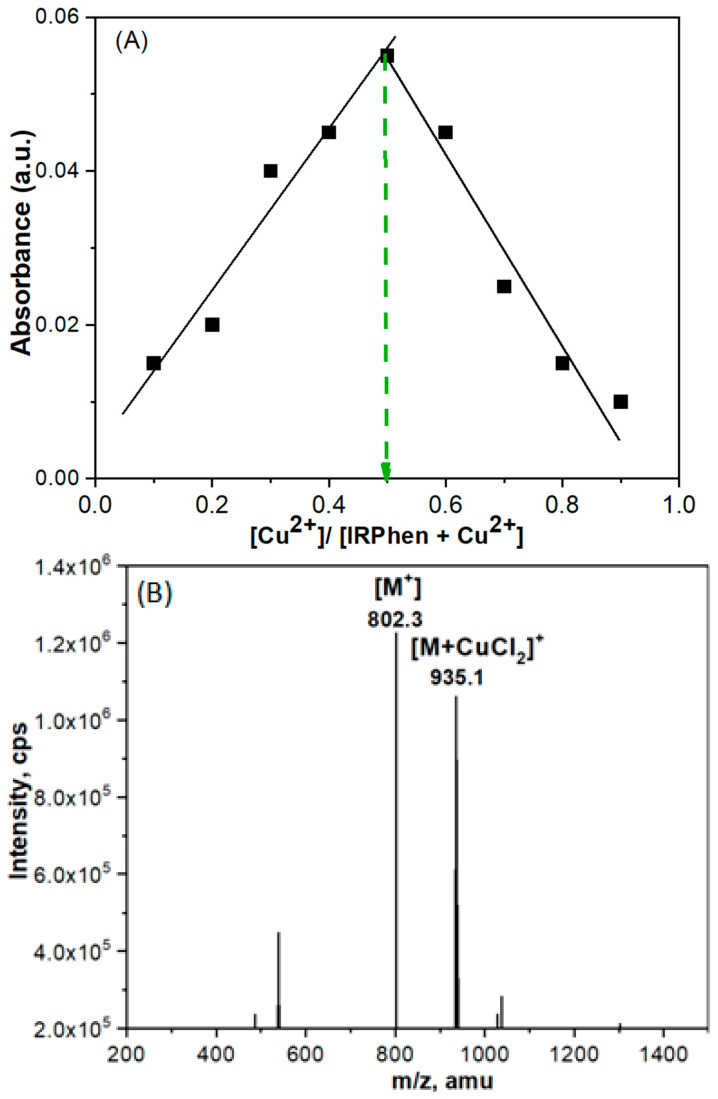
(**A**) Job’s plot of IRPhen and Cu^2+^ in ACN/H_2_O (*v*/*v*, 1:1), showing a maximum absorbance at a molar fraction of 0.5, indicative of a 1:1 binding stoichiometry (Absorbance intensities were recorded at 557 nm). (**B**) ESI-Mass spectrum of the IRPhen–Cu^2+^ complex, showing peaks at *m/z* = 802.3 ([IRPhen]^+^) and *m/z* = 935.1 ([IRPhen + CuCl_2_]^+^), confirming the formation of a 1:1 IRPhen–Cu^2+^ complex.

To investigate the binding stoichiometry between IRPhen and Cu^2+^, both Job’s plot and UV-Vis titration experiments were conducted. As shown in Figure 4A, the Job’s plot exhibited a maximum absorption intensity at 557 nm when the molar fraction of IRPhen was approximately 0.5, indicating a 1:1 stoichiometry between IRPhen and Cu^2+^. This conclusion was further supported by UV–Vis titration studies. As shown in the inset in Figure 1B, the absorbance changes at both 557 nm and 771 nm reached a plateau when the molar ratio of Cu^2+^ to IRPhen approached 1:1, confirming the formation of a 1:1 IRPhen-Cu^2+^ complex.

The binding constant (K) between Cu^2+^ and IRPhen was determined from the absorbance data at 557 nm using a previously reported method [49,50]. The calculated association constant was1.3×10^6^ M^−1^ (log K= 6.11), indicating a strong binding affinity of IRPhen for Cu^2+^.

**Scheme 2:**
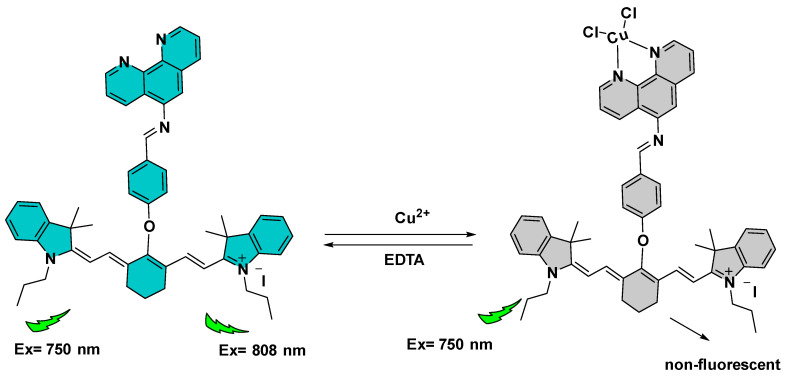
Proposed reversible binding mechanism between IRPhen and Cu^2+^.

Further evidence for complex formation was obtained from ESI-MS analysis. As shown in Figure 4B, the ESI-MS spectrum displayed two prominent peaks: one at *m*/z = 802.3, corresponding to the IRPhen cation (*m*/*z* = 802.3, [IRPhen]^+^), and the other at *m*/*z* = 935.1 corresponding to the 1:1 IRPhen-Cu^2+^ complex with two chloride counterions (*m*/*z* = 935.1, [IRPhen+CuCl_2_]^+^). These results provide compelling evidence for the 1:1 complexation between IRPhen and Cu^2+^. A proposed structure of the IRPhen-CuCl_2_ complex is shown in Scheme 2.

### 3.5. Reversibility and pH Effects on the Binding Between IRPhen and Cu^2+^

**Figure 5:**
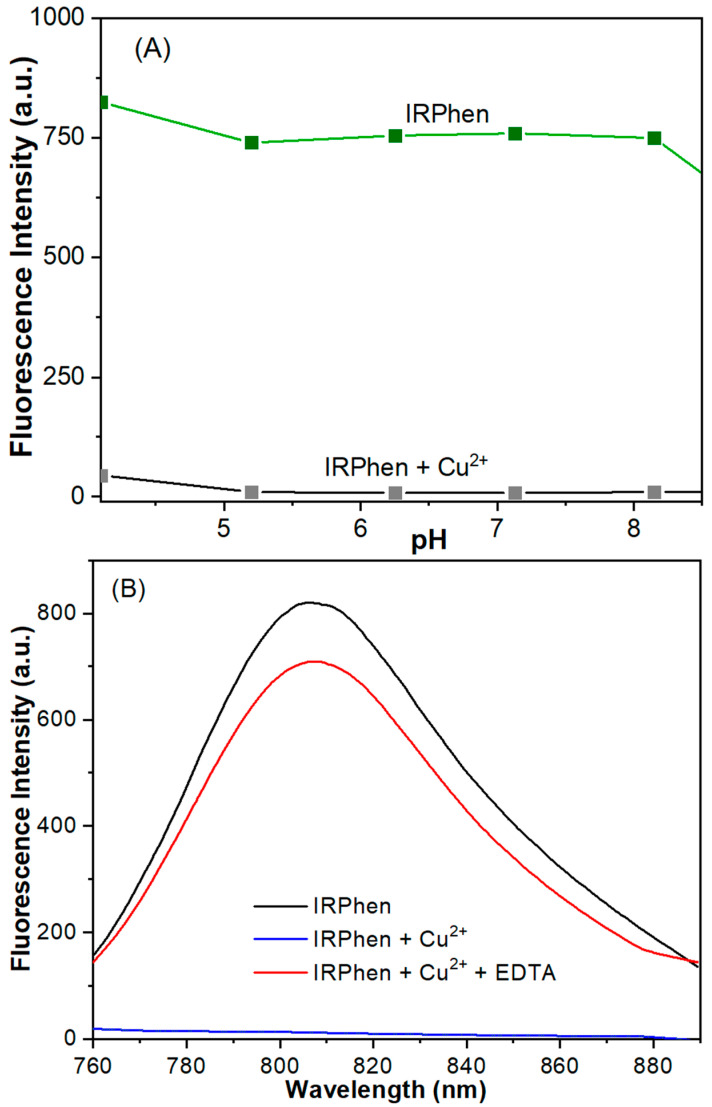
(**A**) Variation in fluorescence intensity at 808 nm for IRPhen (5 µM) and the IRPhen–Cu^2+^ complex (5 µM) over the pH rage 4.0 to 8.2. (**B**) Reversible fluorescence emission response of IRPhen (5 μM) toward Cu^2+^ ions upon subsequent addition of EDTA (5.0 equiv.).

pH is one of the most critical factors influencing the performance of fluorescent sensors in biological systems. To evaluate the pH stability of the IRPhen sensor, fluorescence spectra were recorded over pH values between 4.0 to 8.2, adjusted using dilute HCl. As shown in Figure 5A, the fluorescence intensity of both free IRPhen and the IRPhen–Cu^2+^ complex remained essentially unchanged across the pH range 4.0 to 8.2, demonstrating that the probe is stable under physiological conditions and even at mildly acidic pH (down to pH 4), covering the biological pH range.

The reversibility of the binding between IRPhen and Cu^2+^ was further examined by adding EDTA (5.0 equiv.) to the IRPhen–Cu^2+^ complex solution. Upon EDTA addition, the fluorescence emission at 808 nm was significantly restored (Figure 5B), confirming that Cu^2+^ coordination with IRPhen is reversible. This reversibility suggests that the sensor can potentially be reused and is suitable for dynamic Cu^2+^ monitoring in biological systems. The proposed reversible binding mechanism is illustrated in Scheme 2.

### 3.6. Cell Imaging Studies

**Figure 6:**
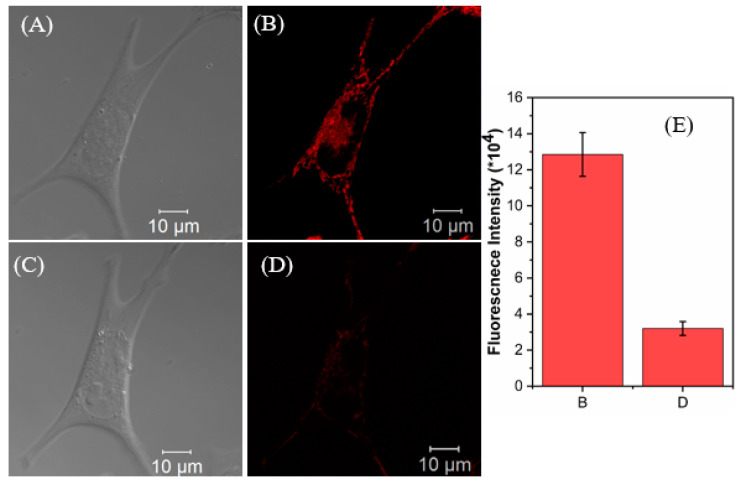
Confocal microscopy images of WS1 fibroblast cells. Bright-field image (**A**) and confocal fluorescence image (**B**) of WS1 cells incubated with IRPhen (10 µM) for 30 min. Bright-field image (**C**) and confocal fluorescence image (**D**) of cells pretreated with Cu^2+^ (10 µM) for 8 h, followed by incubation with IRPhen (10 µM) for 30 min; (**E**) Quantitative bar graph showing the corresponding fluorescence intensities of panels (**B**,**D**). Excitation was at 633 nm, and emission was collected from 650–850 nm. Scale bar, 10 µm.

Motivated by the above results, the ability of IRPhen to detect Cu^2+^ in living fibroblast (WS1) cells was investigated using a confocal microscope (Zeiss LSM 710).After reaching approximately 70% confluency, the cells were transferred to fresh serum-free medium and incubated with IRPhen (10 µM) for 30 min at 37 °C.The cells were then subjected to fluorescence imaging. Since the longest excitation wavelength of our confocal system (Zeiss LSM 710) is 633 nm which lies at the red/near-infrared boundary, IRPhen was excited at 633 nm with emission collected from 650–850 nm for cellular imaging experiments. As shown in Figure 6B, the confocal fluorescence image of WS1 cells stained with IRPhen displayed strong NIR fluorescence signals inside the WS1 cells, indicating that the sensor IRPhen is cell-permeable and capable of functioning effectively in a biological environment.

For Cu^2+^ co-incubation experiments, WS1 cells were first treated with 10 µM CuCl_2_ for 8 h, followed by incubation with 10 µM IRPhen for 30 min prior to confocal imaging. The cells remained viable under the imaging conditions and exhibited normal, healthy morphology when incubated with copper and the sensor (10 µM) (Figure 6A,C), indicating that the sensor is well tolerated and does not induce cytotoxicity under the conditions used. As shown in Figure 6B,D,E, a dramatic decrease in fluorescence intensity was observed after Cu^2+^ co-incubation, consistent with the fluorescence quenching caused by Cu^2+^ binding. These results demonstrate that IRPhen can effectively detect Cu^2+^ and monitor its dynamic changes in living cells.

### 3.7. Comparison with Previously Reported NIR Cu^2+^ Sensors

The sensing performance of the present sensor, IRPhen, toward Cu^2+^ was compared with that of previously reported sensors, as summarized in Table 1. This comparison clearly demonstrates that IRPhen offers several notable advantages, including a low detection limit, excitation and emission wavelengths that both fall within the true NIR region, and reversible binding—an essential feature for monitoring dynamic changes in Cu^2+^ levels.

## 4. Conclusions

In summary, we have developed a novel near-infrared optical sensor, IRPhen, based on a heptamethine cyanine platform conjugated with a 1,10-phenanthroline Cu^2+^-binding receptor. The sensor exhibits strong NIR absorption and emission (E_x_: 750 nm, E_m_: 808 nm), high sensitivity, and good selectivity toward Cu^2+^ over a wide range of competing metal ions. Spectroscopic studies revealed a rapid and reversible 1:1 binding interaction between IRPhen and Cu^2+^, with a high binding affinity (K = 1.3 × 10^6^ M^−1^) and detection limit of 0.286 µM. The probe showed fast response, robust stability across physiological pH ranges, and maintained its sensing properties in competitive environments. Importantly, IRPhen proved to be cell-permeable and capable of monitoring dynamic changes in Cu^2+^ in living fibroblast (WS1) cells using confocal microscopy.

Overall, this sensor design strategy provides a foundation for developing novel NIR probes, offering promising tools for real-time, non-invasive detection of Cu^2+^ in environmental and biological systems, with potential applications in monitoring copper homeostasis and investigating copper-related physiological and pathological processes.

## Figures and Tables

**Table 1:** Comparison of a few reported NIR probes for the detection of Cu^2+^.

Probe	Solvent System	Excitation/Emission Wavelength (nm)	Reversible Binding	LOD (µM)	Bioimaging Cell Models	Reference
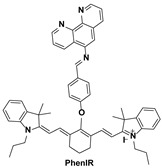	ACN/H_2_O (1/1, *v*/*v*)	E_x_ = 750 nm, E_m_ = 808 nm	Yes	0.286	Living cells/WS1	This work
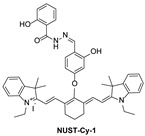	THF/H_2_O (6/4, *v*/*v*)	E_x_ = 450 nm, 750 nm E_m_ = 593 nm, 825 nm	No	1.4	Living cells/HeLa	[27]
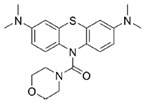	ACN/H_2_O (4/6, *v*/*v*)	Ex = 620 nm E_m_ = 689 nm	No	0.33	Living cells/HepG-2	[33]
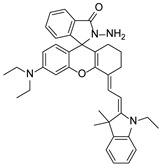	EtOH/PBS (2/8, *v*/*v*)	E_x_ = 640 nm, E_m_ = 730 nm	No	0.362	Living cells/THP-1	[51]
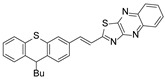	ACN	E_x_ = 670 nm, E_m_ = 975 nm	No	4.44	---	[52]
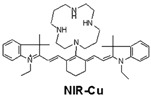	ACN/HEPES (1/4, *v*/*v*)	E_x_ =533 nm, 720 nm, E_m_ = 636 nm, 778 nm	ND	0.089	Living cells/SMMC7721	[53]
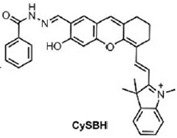	ACN/PBS (1/1, *v*/*v*)	E_x_ = 690 nm, E_m_ = 744 nm	No	0.028	Living cells/A549, Hela	[35]
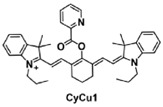	HEPES/DMSO/ CrEL (99/1/0.1, *v*/*v*)	E_x_ = 523 nm, 710 nm E_m_ = 600 nm, 810 nm	No	0.053	Living cells/SH-SY5Y	[25]
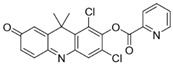	HEPES/DMSO (99.5/0.5, *v*/*v*)	E_x_ = 602 nm, E_m_ = 662 nm	No	0.0018	Living cells/PC-12	[32]
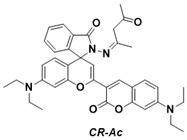	ACN/MOPS (1/1, *v*/*v*)	E_x_ = 425 nm, 650 nm, E_m_ = 520 nm, 696 nm	Yes	0.020	Living cells/WS1	[36]

## Data Availability

Data are contained within the article and are available on request from the corresponding author.
